# Digital health platforms expand access and improve care for male androgenetic alopecia

**DOI:** 10.1111/ijd.16452

**Published:** 2022-10-17

**Authors:** Peter C. Young, Chetan Mahajan, Jerry Shapiro, Antonella Tosti

**Affiliations:** ^1^ Keeps Medical Group New York NY USA; ^2^ Thirty Madison New York NY USA; ^3^ The Ronald O. Perelman Department of Dermatology New York University Grossman School of Medicine New York NY USA; ^4^ Department of Dermatology and Cutaneous Surgery University of Miami Miami FL USA

## Abstract

**Introduction:**

Digital health solutions, specifically direct‐to‐consumer platforms, have been touted to make care accessible and convenient for patients. The aim of this article is to evaluate patients' reported reasons for choosing a platform for the treatment of hair loss and their experience. This platform (Keeps) is focused exclusively on the treatment of male pattern hair loss (MPHL) with approved medications such as oral finasteride and topical minoxidil.

**Methods:**

In order to evaluate patients' motivations to choose the platform and their experience, we administered two distinct questionnaires, with a total of 8983 respondents.

**Results:**

The results showed that patients on the platform report positive health outcomes at approximately 6 months, as 81% of respondents report hair regrowth or cessation of hair loss, and 91% never or rarely miss their medication. Additionally, the platform is expanding the market through education and awareness, as nearly 1 in 3 new patients, mostly in their 20s and early 30s, had never considered treating their hair loss before learning about this virtual care model.

**Conclusion:**

Such a model provides further insights into how digitally enabled care focused on a chronic condition, backed by quality and evidence‐based initiatives, can expand access and improve care for androgenetic alopecia (AGA).

## Introduction

Over 50% of men suffer from male pattern hair loss (MPHL), also known as androgenetic alopecia (AGA). Medical treatments such as finasteride 1 mg and 5% topical minoxidil have been found to be effective in halting, and in some cases reversing, the effects of MPHL.^1^, ^2^, ^3^, ^4^ In recent years, these FDA‐approved treatments have become increasingly available through direct‐to‐consumer platforms, which combine the convenience of virtual care with the delivery of medications directly to the patient's doorstep.

Digital health solutions, specifically direct‐to‐consumer platforms that provide prescription medications after online consultation are expanding in the US.^5^ The emergence of different platforms (Table 1), with Hims, Roman, and Keeps all launching within 2017–2018, has created an offering that is attractive to patients.

**Table 1:** Information gathered from the respective company websites

Company	Year launched	Conditions treated	Services offered	Hair loss/hair care products offered
Blink Health	2015	Men's hair loss, erectile dysfunction, cold sores, cholesterol	Online consult, medication delivery	Finasteride (oral)
Goodrx	2011	Men's hair loss, erectile dysfunction, premature ejaculation, herpes, cold sores, and skin care, among others	Online consult, medication delivery	Finasteride (oral)
His/hers	2017	Hair loss (men and women), erectile dysfunction, premature ejaculation, herpes, skin care, primary care, mental health, general health (supplements)	Online consult, medication delivery, ongoing care	Finasteride (oral), minoxidil (solution), biotin gummies, thickening shampoo, and conditioner
Keeps	2018	Men's hair loss	Online consult, medication delivery, ongoing care	Finasteride (oral), minoxidil (solution and foam), ketoconazole 2%, thickening shampoo, and conditioner
Lemonaid Health	2013	Men's hair loss, erectile dysfunction, premature ejaculation, herpes, cold sores, and skin care, among others	Online consult, medication delivery, integration with Quest diagnostics	Finasteride (oral)
Roman	2017	Men's hair loss, erectile dysfunction, premature ejaculation, herpes, cold sores, skin care, allergies, general health (supplements)	Online consult, medication delivery, ongoing care	Finasteride (oral), minoxidil (solution), Rx dandruff shampoo
Strut Health	2018	Hair loss (men and women), erectile dysfunction, premature ejaculation, herpes, cold sores, skin care	Online consult, medication delivery, ongoing care	Finasteride (oral and topical), dutasteride, vitamins

The aim of this paper is to evaluate patients' motivations for choosing an overall experience with the largest direct‐to‐consumer platform providing treatment for MPHL, Keeps, which has over 300,000 active subscribers across the U.S. as of August 2021.

The platform care team includes board‐certified dermatologists, internal medicine and family medicine physicians, and customer care specialists/coordinators. Quality of care is monitored by the medical director, a board‐certified dermatologist, who reviews a random sample of 2% of each physician's cases. Review audits of over 2000 cases thus far have demonstrated 100% concordance with the diagnosis of MPHL among patients. The medical director also provides ongoing education to the participating physicians and guidance on complex cases as needed.

The patient journey starts via an asynchronous consultation with a physician, which includes scalp photos submitted by the patient. If the diagnosis of MPHL is confirmed, the physician provides a prescription for finasteride 1 mg and/or a recommendation for over‐the‐counter 5% topical minoxidil. The patient is advised to seek an in‐person visit with a dermatologist in case of doubts about the diagnosis or if he is deemed not a match for the available treatments offered by the platform.

Patient enrollment for finasteride and/or minoxidil subscription creates automated touchpoints to ensure the treatment has been initiated, and educational materials are provided throughout the patient's journey. This includes information on anticipated timelines for improvement and potential side effects of the treatment. The platform also utilizes a progress tracker tool, which enables patients to upload follow‐up photos periodically during their treatment. The treating physician reviews these photos and provides feedback to the patient regarding his progress and any recommended treatment modifications. Patients also have the ability to send questions to their physicians throughout the course of treatment. These various touchpoints through the virtual care platform, combined with the delivery of medications directly to the patient's doorstep, provide convenient end‐to‐end access to care and aim to enhance patient engagement and adherence to treatment.

## Materials and methods

Two questionnaires were administered via SurveyMonkey to collect self‐reported data from patients at different points in their treatment journey. Responses for Questionnaire A were collected from August 27, 2020, to May 12, 2021. Questionnaire A is sent to every customer with a non‐canceled account 20 days after they place their 3rd order (i.e., at 200 days on the platform), as treatments typically take at least 4–6 months to demonstrate results for patients. A separate questionnaire (Questionnaire B) was administered to every customer who completed a consultation, received a prescription from a doctor, and whose order has been shipped (i.e., 3 days post‐purchase). Responses for Questionnaire B were collected from December 21, 2020, to February 24, 2021. The list of questions and responses received for both questionnaires are captured in Tables 2 and 3.

**Table 2:** Questionnaire A. Administered to every customer with a non‐canceled account 20 days after they place their 3rd order (i.e., at 200 days on the platform). Responses for Questionnaire A were collected from August 27, 2020, to May 12, 2021, with 3359 responses received out of 64,567 surveys sent (response rate: 5.2%)

	N of Respondents	% of Respondents	95% CI
How would you describe your results with Keeps so far?			
I've had a lot of hair growth	268	8%	7–9%
I have not seen results	654	19%	18–21%
My hair loss has stopped, but I have not had any additional hair growth	962	29%	27–30%
I've had some hair growth	1475	44%	42–46%
How often are you missing your medication?			
I miss it 50% of the time or more	52	1.54%	1–2%
I miss taking it 2–3 times per week	255	7.59%	7–9%
I never miss taking my medication	1475	43.91%	42–46%
I miss it rarely (once per week or less)	1577	46.9%	45–49%

% is more than 100%.

**Table 3:** Questionnaire B. Administered to every customer who completes a consultation, receives a prescription from a doctor, and whose order has been shipped (i.e., 3 days post‐purchase). Responses for Questionnaire B were collected from December 21, 2020, to February 24, 2021, with 5624 responses received out of 34,441 surveys sent (response rate: 16.3%)

Reasons you chose to pursue treatment at Keeps rather than a traditional medical practice (primary and secondary reasons, aggregated)	N of Respondents	% of Respondents	95% CI
Keeps is more convenient for me because everything is online	3378	60%	59–61%
Keeps is more convenient for me because treatment is shipped directly to me	3068	55%	53–56%
I actually had not thought about treating my hair loss until I learned about Keeps	1803	32%	31–33%
I was impressed by the strong reputation of Keeps	1513	27%	26–28%
The price for medications at Keeps is less expensive	1400	25%	24–26%
The price for the consultation with a clinician at Keeps is less expensive	1085	19%	18–20%
I feel safer receiving my care virtually during the covid pandemic	981	17%	16–18%
I was embarrassed/self‐conscious to speak with a clinician about my hair loss in person	974	17%	16–18%
Other (please specify)	386	7%	6–8%

## Results

For Questionnaire A, 3359 responses were received out of 64,567 surveys sent (response rate: 5.2%). A sample of 3359 respondents represents 243,261 patients who have stayed on the platform for at least 200 days. At a 95% level of confidence, the margin of error is 1.7%. Given a 100% completion rate for each reported question on Questionnaire A, the margin of error for each individual question is the same as that of the survey at 1.7% at a 95% level of confidence.

For Questionnaire B, 5624 responses were received out of 34,441 surveys sent (response rate: 16.3%). A sample of 5624 respondents represents 533,614 patients on our platform who have made at least one purchase. At a 95% level of confidence, the margin of error is 1.3%. Given an upwards of 99% completion rate for each reported question on Questionnaire B, the margin of error for each individual question is the same as that of the survey at 1.3% at a 95% level of confidence.

For Questionnaire A, approximately 81% of respondents report positive results, of which 64% report at least some hair regrowth and 36% report cessation of hair loss. Meanwhile, 91% of respondents report never or rarely missing (once per week or less frequently) their medication.

Additionally, through Questionnaire B, we found that the platform is expanding the market through education and awareness, as 32% of new patients surveyed, predominantly in the 20 to early 30‐year‐old range, had never considered treating their hair loss before learning about Keeps. The ability to engage patients at an earlier stage of their hair loss journey bodes well for long‐term outcomes.

## Conclusion

In this paper, we have focused on direct‐to‐consumer platforms for treating MPHL, but many similar platforms exist across a variety of other dermatologic conditions, such as acne and anti‐aging. Access to dermatologic care in the U.S. has been a challenge for many years, based on the overwhelming demand of patients compared to the supply of dermatologists. The utilization of mid‐level dermatology providers under the supervision of dermatologists has only partially filled this gap. The use of virtual care with technology‐driven processes provides an immense opportunity to deliver accessible dermatologic care at a large scale.

Direct‐to‐consumer platforms have gained traction over the last several years, with acceleration in the past year due to the Coronavirus pandemic. Telemedicine is an effective option for diagnosing and treating hair disorders, and many patients have felt safer engaging in telehealth visits than in‐person visits throughout the pandemic.^6^ It has been anticipated that the convenience of virtual care will continue to drive the increased popularity of this modality over the coming years.^7^ This is particularly true for the tech‐savvy millennial and Gen‐Z populations, who increasingly seek on‐demand care for a wide variety of medical conditions.

The results of this study show the ability of a virtual care platform to extend evidence‐based MPHL treatments to a large patient population, which includes a significant portion of patients who had not considered treating their hair loss until hearing about the platform. Additionally, the results demonstrated the virtual care platform's ability to promote treatment adherence and deliver favorable treatment outcomes for its patients. This study also highlights the importance of dermatologists providing rigorous oversight of direct‐to‐consumer telehealth platforms providing care for dermatologic conditions. This should include robust quality improvement programs, e.g., chart audits, to ensure that high‐quality evidence‐based dermatologic care is provided.

While there remain pockets of skepticism and resistance toward direct‐to‐consumer telehealth in the medical community, consumerism will inevitably catalyze further adoption of this model. The dermatology community should continue to monitor the progress of direct‐to‐consumer telehealth platforms, as these may offer important lessons for improving access to high‐quality dermatologic care. In addition to standalone direct‐to‐consumer telehealth companies, there are opportunities to integrate this model into existing dermatology practices in order to improve access and the overall patient experience.

The purpose of this study is not to compare online platforms vs in‐person consultations for male androgenetic alopecia but to evaluate the satisfaction of patients who have already chosen the online option and to understand their reasons.

Although in‐person evaluation by a dermatologist, including trichoscopy, remains the gold standard for managing male androgenetic alopecia, this study has shown that virtual consultation, if properly organized and monitored, may provide a viable alternative. In addition, the use of teletrichoscopy and teletrichology is a promising new option to interact with hair disorder patients remotely and, if properly performed, is effective in selecting patients who need in‐person consultation for diagnostic or therapeutic procedures.^6^

Patients' approach to their medical care has dramatically changed in the last few years, with the increasing role of social media and online medical providers. New generations clearly want to play a more active role in the choice of their medical care. We believe it is important for dermatologists to understand the new online and virtual healthcare options, as well as patient preferences and motivations that drive their decisions when seeking medical care and advice.

### Limitations

There is a possibility of non‐response bias as patients who chose not to respond to questionnaire A could differ in their satisfaction from those who responded. However, since all patients who were sent the questionnaire needed to have placed multiple orders and have been active on the platform for over 6 months, it is reasonable to assume that non‐respondents are satisfied enough to continue with their treatment. A similar limitation of non‐response bias applies to questionnaire B as well. However, since questions of interest centered around attitudes toward Keeps vs. traditional medical practice, and we report attitudes of patients who ultimately purchased a Keeps product, it is likely that respondents would be similar to non‐respondents.
